# Heterologous expression reveals a cryptic morphogenetic lanthipeptide durhapeptin from *Streptomyces durhamensis*

**DOI:** 10.1007/s10482-026-02378-8

**Published:** 2026-07-18

**Authors:** Marino Tsugimoto, Ryo Kobayashi, Chanaphat Thetsana, Tomohiro Furukawa, Hiroyuki Nakagawa, Shinya Kodani

**Affiliations:** 1 Graduate School of Integrated Science and Technology, Shizuoka University, 836 Ohya, Suruga-ku, Shizuoka, 422-8529 Japan; 2 Graduate School of Science and Technology, Shizuoka University, 836, Ohya, Suruga-ku, Shizuoka, 422-8529 Japan; 3 Institute of Food Research, National Agriculture and Food Research Organization (NARO), 2-1-12 Kannondai, Tsukuba, Ibaraki 305-8642 Japan; 4 Research Center for Advanced Analysis, Core Technology Research Headquarters, NARO, 2-1-12 Kannondai, Tsukuba, Ibaraki 305-8642 Japan; 5 College of Agriculture, Academic Institute, Shizuoka University, 836 Ohya, Suruga-ku, Shizuoka, 422-8529 Japan

**Keywords:** Heterologous production, Lanthipeptide, Morphogen, *Streptomyces durhamensis*

## Abstract

Morphological differentiation in *Streptomyces* is regulated by extracellular peptide signals, including class III lanthipeptides such as SapB and AmfS. Genome mining of *Streptomyces durhamensis* identified a previously uncharacterized class III lanthipeptide biosynthetic gene cluster. The precursor peptide and the modifying enzyme were heterologously expressed in *Escherichia coli*, enabling the production of a lanthipeptide designated durhapeptin. Mass spectrometric analyses combined with partial hydrolysis supported the presence of two labionin cross-links in the mature peptide. Functional assays demonstrated that a durhapeptin variant selectively induced aerial hyphae formation in several *Streptomyces* species harboring related gene clusters, whereas no activity was observed in the original producer strain or in distantly related species. Substitution of hydrophobic residues abolished morphogenetic activity, indicating that hydrophobicity is critical for its biological function. These findings suggest that cryptic class III lanthipeptides contribute to species-specific regulation of morphological differentiation and highlight the importance of this group of lanthipeptides in the developmental biology of streptomycetes.

**Supplementary information:**

The online version contains supplementary material available at 10.1007/s10482-026-02378-8.

## Introduction

Lanthipeptides are a large family of ribosomally synthesized and post-translationally modified peptides that display diverse biological functions, including antimicrobial activity and the regulation of microbial development (Garcia-Ausencio et al. 2025; Lee and van der Donk 2022; Repka et al. 2017). Their defining structural features arise from enzyme-mediated modifications of precursor peptides, in which selected serine, threonine, and cysteine residues in the core region are converted into thioether-linked amino acids such as lanthionine (Lan) and methyllanthionine (MeLan). These transformations involve dehydration of Ser/Thr residues to form dehydroamino acids (dehydroalanine, Dha; dehydrobutyrine, Dhb), followed by intramolecular cyclization with cysteine thiols to generate Lan or MeLan, and culminate in proteolytic removal of the leader peptide to release the mature product. Based on the nature of the modification enzymes, lanthipeptides are classified into five major classes I-V (Ding et al. 2024; Pei et al. 2022). Among them, class III lanthipeptides are notable for their structural diversity and, in some members, for the presence of the unusual tricyclic amino acid labionin (Lab). Representative examples include the labyrinthopeptins from *Actinomadura namibiensis*, in which Lab is formed through LabKC-catalyzed linkage of a single cysteine residue to two dehydroalanine residues (Krawczyk et al. 2013; Meindl et al. 2010). To identify novel and previously unexplored lanthipeptides, genome mining approaches have been widely applied. This strategy entails systematic screening of large-scale bacterial genome databases to detect biosynthetic gene clusters associated with lanthipeptide biosynthesis. Using this approach, approximately 8,500 genes encoding lanthipeptide precursor peptides have been identified in more than 100,000 bacterial and archaeal genomes (Walker et al. 2020). Since lanthipeptides are secondary metabolites, they are typically not produced under standard laboratory culture conditions. Therefore, heterologous expression of their biosynthetic genes is an efficient strategy for lanthipeptide production. To date, several class III lanthipeptides, including labyrinthopeptin (Krawczyk et al. 2013), curvopeptin (Jungmann et al. 2014; Krawczyk et al. 2012; Voller et al. 2012), stackepeptin (Jungmann et al. 2016), catenulipeptin (Wang and van der Donk 2012), flavipeptin (Voller et al. 2013), noursin (Li et al. 2023), rochsin (Liu et al. 2024) have been produced through heterologous expression guided by genome mining approaches.

Several class III lanthipeptides have been implicated in the morphological development of streptomycetes. *Streptomyces coelicolor*, a model organism for bacterial differentiation, undergoes a complex life cycle involving the formation of aerial hyphae and spores, a process that is regulated in part by extracellular peptide signals (Willey et al. 1991, 1993, 2006). The lanthipeptides SapB and AmfS are well-characterized morphogens that promote aerial mycelium formation in *S. coelicolor* and *S. griseus* (Kodani et al. 2004; Takano et al. 2017; Ueda et al. 2002; Willey et al. 1991). Based on their ring composition, class III lanthipeptides can be broadly divided into three groups: peptides containing only Lan/MeLan, those containing Lab, and those possessing a mixture of these structural elements (Hegemann and Sussmuth 2020). Although biosynthetic gene clusters (BGCs) for these peptides are widely distributed among streptomycetes, morphogenetic activity has been demonstrated for only a limited number of them.

In this context, we investigated a previously uncharacterized class III lanthipeptide BGC from *S. durhamensi*s and examined its product through heterologous expression, leading to the identification and functional characterization of novel lanthipeptides designated durhapeptins.

## Materials and methods

### Bacterial strains

For molecular cloning, chemically competent *Escherichia coli* DH5α cells (SMOBIO Technology, Inc., Taiwan) were used. For heterologous gene expression, competent *E. coli* BL21(DE3) cells (New England Biolabs, Ipswich, MA, USA) were employed as the expression host. The following bacterial strains were obtained from the NITE Biological Resource Center (NBRC, Japan): *Streptomyces filipinensis* NBRC12860^T^, *Streptomyces humidus* NBRC12877^T^, *Streptomyces durhamensis* NBRC13441^T^, *Streptomyces alanosinicus* NBRC13493^T^, *Streptomyces djakartensis* NBRC15409^T^, *Streptomyces hygroscopicus* subsp. *hygroscopicus* NBRC16555, *Streptomyces siamensis* NBRC108799^T^, *Streptomyces maremycinicus* NBRC110468^T^, *Streptomyces blastmyceticus* NBRC12747^T^, *Streptomyces hawaiiensis* NBRC12784^T^, *Streptomyces umbrinus* NBRC13091^T^, *Streptomyces asoensis* NBRC13813^T^, *Streptomyces echinoruber* NBRC14238^T^, *Streptomyces cinereospinus* NBRC15397^T^, and *Streptomyces mobaraensis* NBRC13819^T^. The following bacterial strains were obtained from the RIKEN Bioresource Research Center (RIKEN BRC, Japan): *Streptomyces davaonensis* JCM4913^T^, and *Streptomyces viridochromogenes* JCM4437^T^.

### Genome mining of lanthipeptide biosynthetic gene clusters

A sequence similarity network (SSN) analysis of class III lanthipeptide synthetases (LanKCs) was performed as described previously (Kobayashi et al. 2024).

### Molecular cloning of the co-expression of the *durA* and *durKC* genes in *E. coli*

The DNA sequences of *durKC* and *durA* (Fig. S1 and S2) were codon-optimized for expression in *Escherichia coli*. The synthesized gene fragments were inserted between the *NdeI* and *KpnI* restriction sites of the expression vector pET-29b ( +), yielding the construct pET29b-13441. In this construct, the native gene order was rearranged to *durA*–*durKC* primarily to optimize expression efficiency in *E. coli*. Genes closer to the promoter generally show higher expression levels. Since the precursor peptide (DurA) acts as the substrate, it is required in a much higher molar quantity than the modification enzyme (DurKC). Placing *durA* first ensures an abundant pool of precursor peptides for efficient post-translational modification. The plasmid was synthesized by Twist Bioscience (San Francisco, CA, USA). Competent *E. coli* BL21(DE3) cells were transformed with pET29b-13441 by electroporation. Transformants were plated on LB agar supplemented with kanamycin (30 µg/mL) and incubated at 30 °C until colony formation.

### Heterologous production of durhapeptin in the expression host *E. coli*

*E. coli* BL21 (DE3) harboring pET29b-13441 was cultured on modified basal agar medium (Kaweewan et al. 2024) supplemented with kanamycin (30 µg/mL) and isopropyl β-D-thiogalactopyranoside (IPTG; 0.1 mM) for durhapeptin production. The transformant cells were grown on modified basal agar at 30 °C for 24 h and harvested by scraping the agar surface with a sterile spatula. The collected cells were extracted with an equal volume of methanol (w/v) and centrifuged at 4,000 rpm for 15 min. The resulting supernatant was analyzed by high-performance liquid chromatography (HPLC) using a C18 (ODS) column (4.6 × 250 mm; Wakopak Handy ODS, FUJIFILM Wako Pure Chemical Co., Ltd., Japan). The HPLC system employed a two-solvent system consisting of mobile phase A (distilled water containing 0.05% trifluoroacetic acid (TFA)) and mobile phase B (MeCN containing 0.05% TFA) at a flow rate of 1.0 mL/min. UV detection was performed at 220 nm. Isolation of durhapeptin was achieved by repeated HPLC separations using a linear gradient in which mobile phase B was increased from 10 to 100% over 20 min.

### Heterologous production of durhapeptin variant peptides in *E. coli*

The expression vector pET29b-13441-S26T was constructed by site-directed mutagenesis using inverse PCR. Inverse PCR was performed with EmeraldAmp MAX PCR Master Mix (Takara Bio Inc., Japan) using the primer pair 13441-S26T-F and 13441-S26T-R (Table S1) and pET29b-13441 as the template. The amplified product was chemically transformed into *E. coli* DH5α, and plasmid DNA was isolated using the FastGene Plasmid Mini Kit (NIPPON Genetics Co., Ltd., Japan). After sequence confirmation, the validated plasmid pET29b-13441-S26T was transformed into *E. coli* BL21 (DE3) for heterologous expression of the durhapeptin S26T variant. The vectors pET29b-13441-S26T-L34T, pET29b-13441-S26T-L35T, and pET29b-13441-S26T-L37T were constructed by inverse PCR using the corresponding primer sets (Tables S1 and S2) with pET29b-13441-S26T as the template. Additional variants, including pET29b-13441-S26T-L35T-L24T, pET29b-13441-S26T-L35T-L25T, and pET29b-13441-S26T-L35T-V28T, were generated using the appropriate primer sets (Table S2) with pET29b-13441-S26T-L35T as the template. Transformation and heterologous production of each variant peptide were performed as described above for durhapeptin.

### Partial TFA hydrolysis of durhapeptin S26T-L35T-V28T

Durhapeptin S26T-L35T-V28T was dissolved in 500 µL of 2% aqueous trifluoroacetic acid (TFA), and the reaction was conducted at 60 °C for 24 h. The reaction mixture was subsequently subjected to HPLC for fractionation. HPLC conditions were as described above; however, purification was performed using a linear gradient in which mobile phase B (MeCN containing 0.05% TFA) was increased from 33 to 42% over 20 min.

### Mass spectrometry (MS) experiments

Electrospray ionization (ESI) MS analyses were performed using a JEOL JMS-T100LP mass spectrometer. Collision-induced dissociation (CID) mass experiment was performed using an Exactive Orbitrap mass spectrometer (Thermo Fisher Scientific, Waltham, MA, USA) equipped with a heated electrospray ionization (HESI-II) source. Measurements were conducted in positive ion mode over an *m/z* range of 120–2,000. CID was performed using the higher-energy collisional dissociation (HCD) cell, with collision energies set to 0, 40, and 80 eV. High-resolution accurate-mass (HR/AM) spectra were acquired under CID conditions, and fragment ion *m/z* values were used for structural interpretation.

### Aerial hyphae-inducing activity assay

The assay was performed as described previously (Kodani et al. 2005).

### Chemical investigation of the extract of *Streptomyces durhamensis* NBRC 13441

To evaluate lanthipeptide production under native conditions, *Streptomyces durhamensis* NBRC 13441 was cultivated on ISP2 agar medium (Kaweewan et al. 2019) at 30 °C for 7 days. Following cultivation, the cells were harvested and extracted with an equal volume of methanol (w/v). After centrifugation at 14,500 × g for 2 min, the resulting supernatant was subjected to high-performance liquid chromatography (HPLC) analysis. Separation was carried out using a reversed-phase ODS (C18) column (4.6 × 250 mm; Wakopak Handy ODS, FUJIFILM Wako Pure Chemical Co., Ltd., Japan) coupled to a photodiode array detector (MD-2018Plus, JASCO, Japan). UV spectra were recorded over the range of 200–400 nm at a flow rate of 1.0 mL/min. The mobile phase consisted of solvent A (distilled water containing 0.05% TFA) and solvent B (MeCN containing 0.05% TFA), and elution was performed using a linear gradient in which solvent B was increased from 10 to 100% over 20 min.

## Results

### Analysis of a biosynthetic gene cluster of durhapeptin

**Fig. 1:**
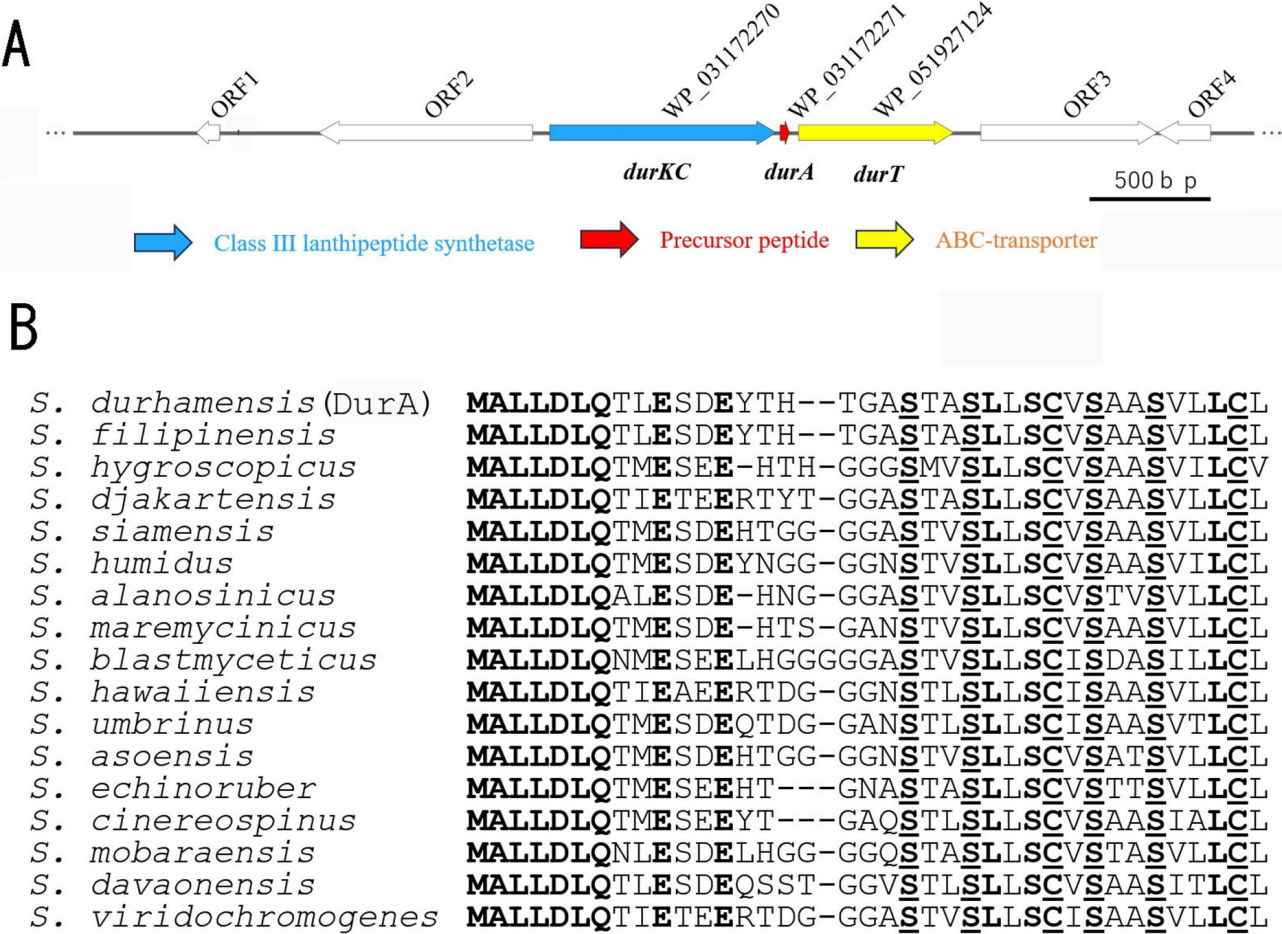
**A** Biosynthetic gene cluster of durhapeptin **B** Alignment of the precursor peptide related to DurA. Bold letters: recognition sequence for lanthipeptide synthetases; Bold with underline: serine and cysteine, which can be involved in lab formation. G-X dotted underlined: a specific cleavage site of protease domain of dual-functional ATP-binding cassette transporter. Overall, these structural and genetic characteristics strongly support the involvement of the *dur* cluster in durhapeptin biosynthesis

**Fig. 2:**
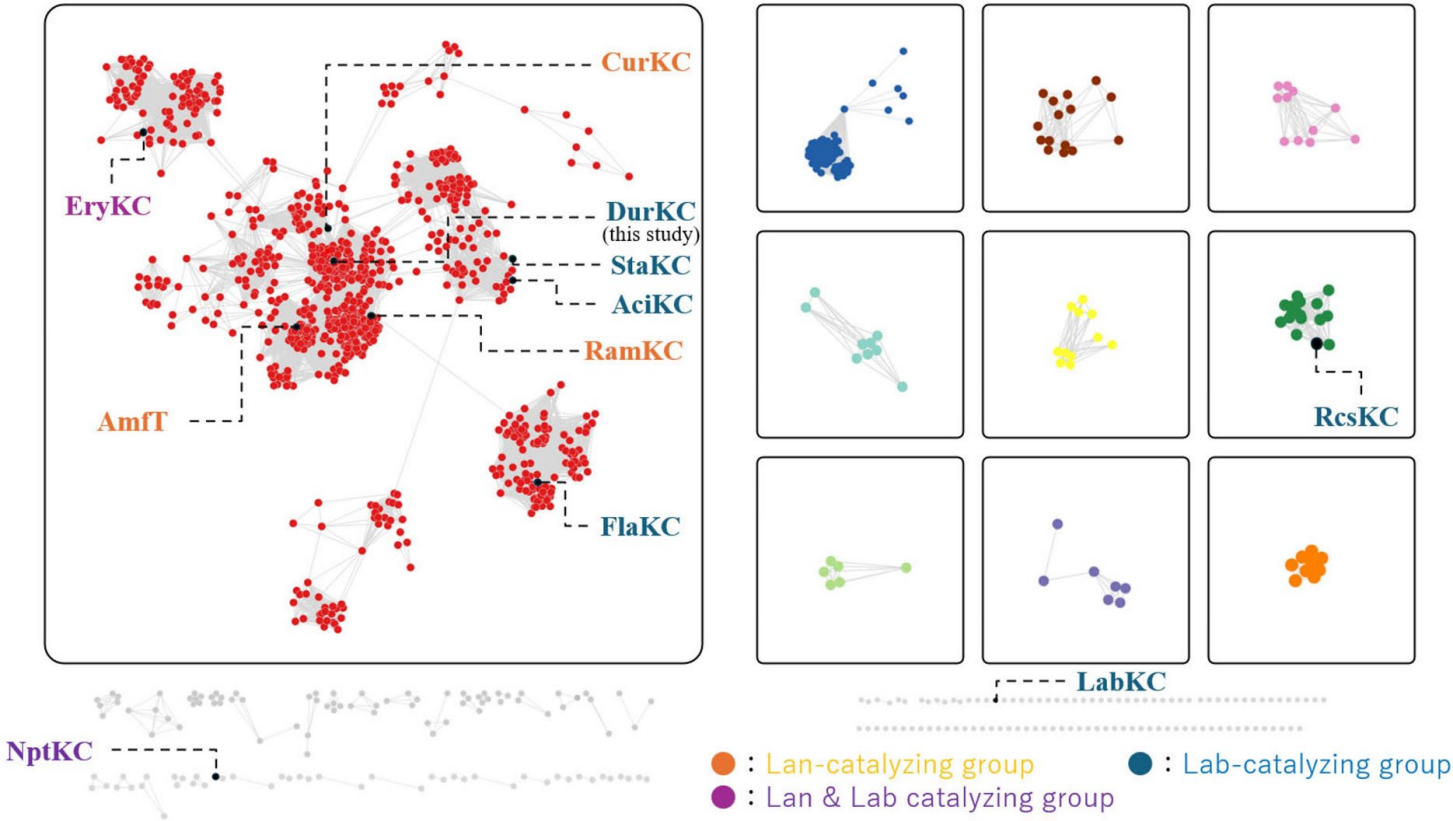
SSN analysis for class III lanthipeptide synthetases related to DurKC. The related genes are indicated with colors; orange letter: the synthetase catalyzing Lan formation, blue letter: the synthetase catalyzing Lab formation, purple letter: the synthetase catalyzing lab and Lan formation. This network analysis successfully clusters DurKC within the group of Lab-forming synthetases

Genome mining has revealed that class III lanthipeptide biosynthetic gene clusters (BGCs) are widely distributed among actinobacterial genomes (Walker et al. 2020). Among these, SapB and AmfS are representative lanthipeptide morphogens (Kodani et al. 2004; Takano et al. 2017; Ueda et al. 2002). In the NCBI database, this group of lanthipeptide-coding genes is annotated as the “SapB/AmfS family lanthipeptide,” which currently comprises more than 4,000 sequences. Among the precursor peptide-coding genes in this group, we identified a well-conserved and highly hydrophobic peptide subgroup by BLASTP analysis (Fig. 1B). Among these, we identified a biosynthetic gene cluster (BGC) in the genome of *Streptomyces durhamensis* consisting of three genes: the precursor peptide gene *durA***,** the lanthipeptide synthetase gene *durKC*, and the transporter gene *durT* (Fig. 1A and Table S1). The leader peptide sequences shared a conserved L–L–D–L–Q motif, which was predicted to form an α-helix by the CRNPRED program (Kinjo and Nishikawa 2006) and to serve as a recognition sequence for DurKC (Wiebach et al. 2020). The core peptide sequences commonly contained two tandem SXXSXXXC motifs, potentially enabling the formation of two Lab/Lan units during biosynthesis (underlined in Fig. 1B). The core biosynthetic genes are flanked by *ORF3* and *ORF4*, which are predicted to encode an ATP-binding cassette (ABC) domain-containing protein and a response regulator transcription factor, respectively (Table S1). Interestingly, the overall genetic organization and sequence characteristics of these five-gene cluster (*durKC, durA, durT, ORF3,* and *ORF4*) exhibit similarities to the canonical morphogenetic systems in streptomycetes, specifically the *ramCSAB* and *ramR* locus of *Streptomyces coelicolor*, as well as the *amfTSAB* and *amfR* locus of *Streptomyces griseus*. This structural conservation strongly supports the involvement of the whole *dur* cluster in the regulation of morphological differentiation. Sequence similarity network (SSN) analysis (Kobayashi et al. 2024; Zallot et al. 2019) using the DurKC sequence grouped DurKC with the known lanthipeptide synthetases RamC and AmfT, which are responsible for the production of SapB and AmfS, respectively (Fig. 2). Despite the sequence similarity among synthetases in this group, the resulting lanthipeptides are diversified into three types-Lan/MeLan-only, Lab-only, and mixed Lan/MeLan/Lab, making it difficult to predict amino acid specificity (Lan/MeLan/Lab) based solely on synthetase sequence (Fig. 2). Chemical analysis of the methanol extract of *S. durhamensis* cells grown on ISP2 agar medium for 7 days at 30 °C did not detect the expected peptide or its potential biosynthetic derivatives under this specific condition. This lack of detectable production prompted us to perform heterologous expression of the *dur* BGC to determine its structure and biological activity.

### Heterologous production of durhapeptin

Codon-optimized synthetic genes were assembled into the expression vector pET29b-13441 (Fig. S1), derived from the commercial vector pET-29b( +), by sequential insertion of *durA* (precursor peptide) and *durKC* (lanthipeptide synthetase) downstream of the T7 promoter (Fig. S2). The resulting plasmid was introduced into *Escherichia coli* BL21(DE3). Cultures were grown at 30 °C for 24 h in modified basal medium supplemented with kanamycin and isopropyl-β-D-thiogalactopyranoside (IPTG). Cells were extracted with methanol, and the extract was purified repeatedly by HPLC (Fig. S3), yielding a novel lanthipeptide, designated durhapeptin. Electrospray ionization mass spectrometry (ESI–MS) revealed a [M + 2H]^2+^ ion at m/z 1083.5, corresponding to an observed molecular mass of 2165.0 Da (Fig. S4). This experimental mass perfectly matches the calculated mass of 2165.03 Da for the core peptide of DurA (TGASTASLLSCVSAASVLLCL; theoretical unmodified monoisotopic mass of 1966.01 Da) that has undergone six dehydration events (–108.06 Da) and a subsequent post-translational glutathionylation (+ 307.08 Da). This confirms that the detected peak is the fully dehydrated durhapeptin core containing a glutathione adduct, a modification commonly generated during heterologous expression in *E. coli*. This observation is consistent with the known propensity of dehydroalanine residues to undergo glutathionylation in vivo (Lee et al. 2023). The core region of *durA* contained five serine and four cysteine residues (underlined in Fig. 1A). Given the presence of two SXXSXXXC motifs, we hypothesized that Ser26 of *durA* was converted to dehydroalanine (Dha) and subsequently conjugated with glutathione in the *E. coli* host.

### Heterologous production of the variant peptide durhapeptin S26T

To prevent glutathionylation, Ser26 was substituted with threonine (S26T). The electron-donating methyl group of threonine increases electron density at the β-position of the resulting dehydrobutyrine (Dhb), rendering its double bond substantially less reactive toward glutathione than that of Dha. Site-directed mutagenesis was performed by inverse PCR (primers 13441-S26T-F and 13441-S26T-R; Table S2) to generate pET29b-13441-S26T. Heterologous expression of the mutated vector afforded durhapeptin S26T (Fig. S5). In positive-mode ESI–MS, a [M + H]^+^ ion was observed at m/z 1909.0 (Fig. S6). The core sequence (TGASTASLLTCVSAASVLLCL; underlined residue indicates the Thr substitution at position 26 has a calculated monoisotopic mass of 1980.0 Da. Thus, the loss of four water molecules (-72 Da) is consistent with DurKC-mediated modification. The *N*-terminal portion of the precursor peptide may have been cleaved by endogenous *E. coli* proteases. Because the sequence contains two SXXSXXXC motifs, four dehydration events are expected during biosynthesis, suggesting that Thr26 itself was not dehydrated. Durhapeptin S26T exhibited poor solubility in H_2_O, MeOH, and acetone, forming aggregates, but was comparatively soluble in DMSO up to ~ 1.0 mg/mL. We inferred that the peptide is highly hydrophobic due to multiple leucine residues and therefore designed additional variants by substituting selected hydrophobic residues with threonine to increase hydrophilicity.

### Production of variant peptides

Additional site-directed mutagenesis using inverse PCR (primer sets listed in Table S2) generated a panel of variants, which were heterologously expressed and analyzed by HPLC and ESI–MS of MeOH extracts. Production outcomes are summarized in Table 1 and Figs. S7-S14. Relative to durhapeptin S26T, substitution of Leu34 or Leu37 with threonine abolished production, whereas substitution of Leu35 with threonine increased yield. Further substitutions at Leu24 and Leu25 on the S26T-L35T background reduced production. Substitution of Val28 with threonine on the S26T-L35T background resulted in high-level production of the expected peptide, durhapeptin S26T-L35T-V28T, which was selected for structural analysis. Produced variant peptides, except for S26T, exhibited a sequence one amino acid shorter at the *N*-terminus (loss of the Thr residue), as inferred from MS data. During heterologous production in *E. coli*, endogenous proteases cleave the *N*-terminal portion of the peptide, and specific amino acid substitutions may alter susceptibility to proteolytic cleavage.

**Table 1:** Production of variant peptides of durhapeptin (DP)

Peptides	Sequence	Production
Durhapeptin (DP)*	TGASTASLLSCVSAASVLLCL	+
DP S26T	TGASTASLLTCVSAASVLLCL	+
DP S26T-L34T	TGASTASLLTCVSAASVTLCL	−
DP S26T-L35T	TGASTASLLTCVSAASVLTCL	+ +
DP S26T-L37T	TGASTASLLTCVSAASVLLCT	−
DP S26T-L35T-L24T	TGASTASTLTCVSAASVLTCL	±
DP S26T-L35T-L25T	TGASTASLTTCVSAASVLTCL	±
DP S26T-L35T-V28T	TGASTASLLTCTSAASVLTCL	+ +

Underlined residue: substitution with Thr, -: no production, + : detected, + + : high production, ± : low production, *: glutathionylated

### Structure determination of durhapeptin S26T-L35T-V28T

**Fig. 3:**
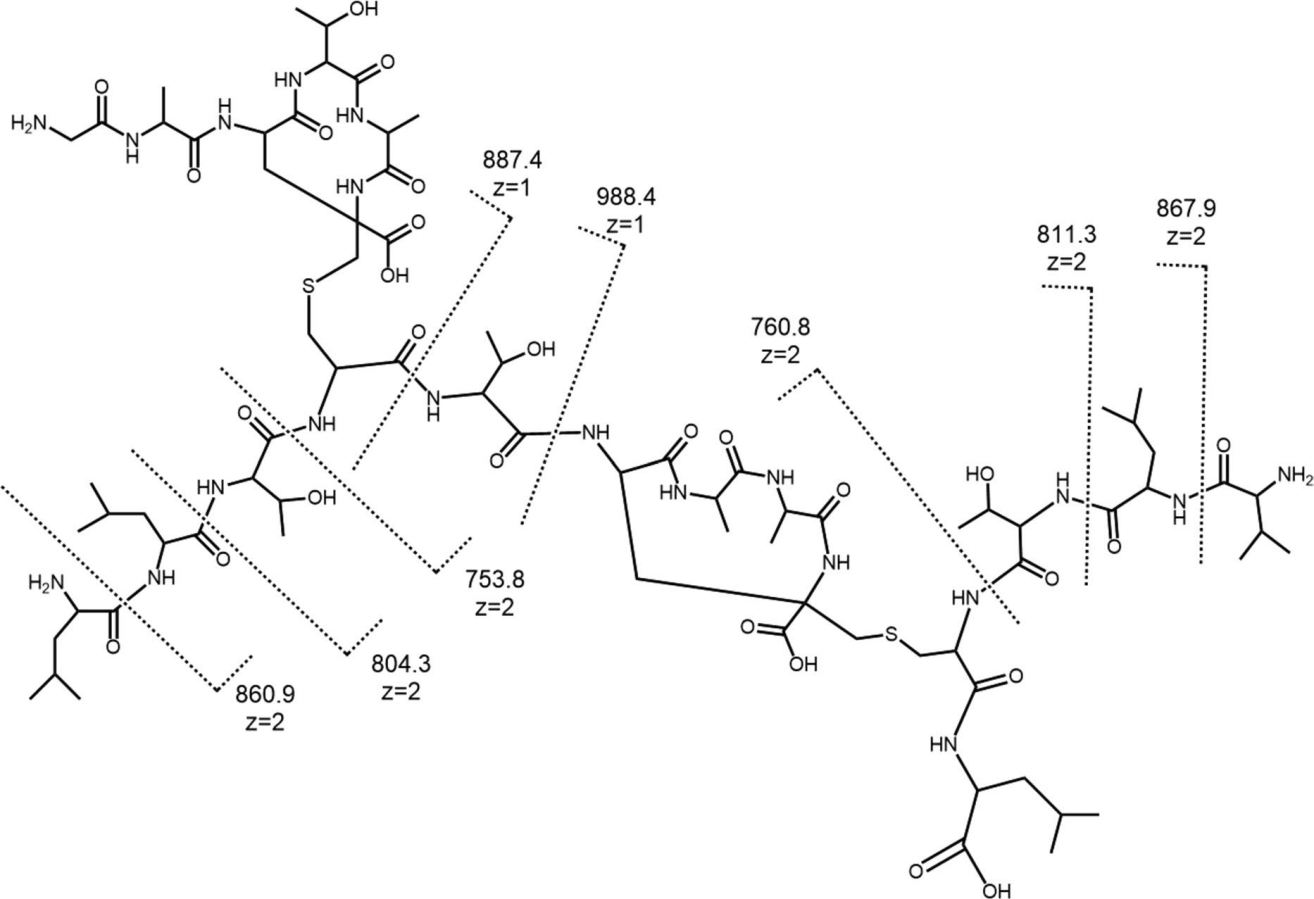
Fragmentations obtained by CID experiments on TFA-treated durhapeptin S26T-L35T-V28T. Observed ions are mapped out along the peptide backbone. The diagnostic fragment ions obtained after partial TFA hydrolysis support the proposed connectivity of the thioether rings in this engineered variant

Attempts to acquire NMR spectra in DMSO-*d*_6_ were hindered by broad ^1^H signals, preventing detailed 2D experiments. Therefore, a chemical treatment was applied to selectively cleave peptide bonds within the Lab rings: mild hydrolysis with trifluoroacetic acid (TFA). Durhapeptin S26T-L35T-V28T was incubated in 2% TFA at 60 °C for 24 h. Following HPLC purification (Fig. S15), ESI–MS analysis indicated a + 36 Da mass shift (Fig. S16), consistent with the addition of two water molecules and cleavage of two peptide bonds within the cyclic regions. CID-MS analysis of the TFA-treated sample (Figs. S17–S21) revealed fragmentation patterns consistent with hydrolysis of two peptide bonds (Fig. 3), supporting a proposed structure containing two Lab rings (Fig. 3, Table S3). By analogy, other durhapeptin variants are likely to possess the same ring topology.

### Aerial hyphae formation-inducing activity of durhapeptin S26T

Because the heterologously produced native durhapeptin was glutathionylated and produced inconsistently, durhapeptin S26T was used for aerial hyphae induction assays (Kodani et al. 2005). Durhapeptin S26T was dissolved in DMSO at 1.0 mg/mL. Its activity was tested against streptomycetes carrying durhapeptin or related BGCs (17 strains listed in Fig. 1B). Spore cells of each strain were spread on ISP2 agar medium; when substrate mycelium had formed (12–48 h), 5 μg of durhapeptin S26T (in DMSO) was spotted onto the lawn. Induction of aerial hyphae around the spot was visually scored after incubation at 30 °C for 24–48 h. Durhapeptin S26T induced aerial hyphae in four strains, *S. humidus*, *S. hygroscopicus*,* S*. *maremycinicus*, and *S. asoensis* (Table S4; Figs. S22–S24), but not in the remaining strains (Fig. 1B). Notably, the original strain, *S. durhamensis*, did not respond to durhapeptin S26T. We also tested durhapeptin S26T against SapB- and AmfS-producing strains (*S. coelicolor* and *S. griseus*) and observed no activity. In contrast, *S. asoensis* showed a marked response. Therefore, the activity of three durhapeptin variants-durhapeptin S26T, durhapeptin S26T-L35T, and S26T-L35T-V28T-was evaluated in *S. asoensis*. Durhapeptin S26T potently induced aerial hyphae formation. In contrast, durhapeptin S26T-L35T and S26T-L35T-V28T lacked activity, indicating that substitution of hydrophobic residues abrogates morphogenetic function.

## Discussion

**Fig. 4:**
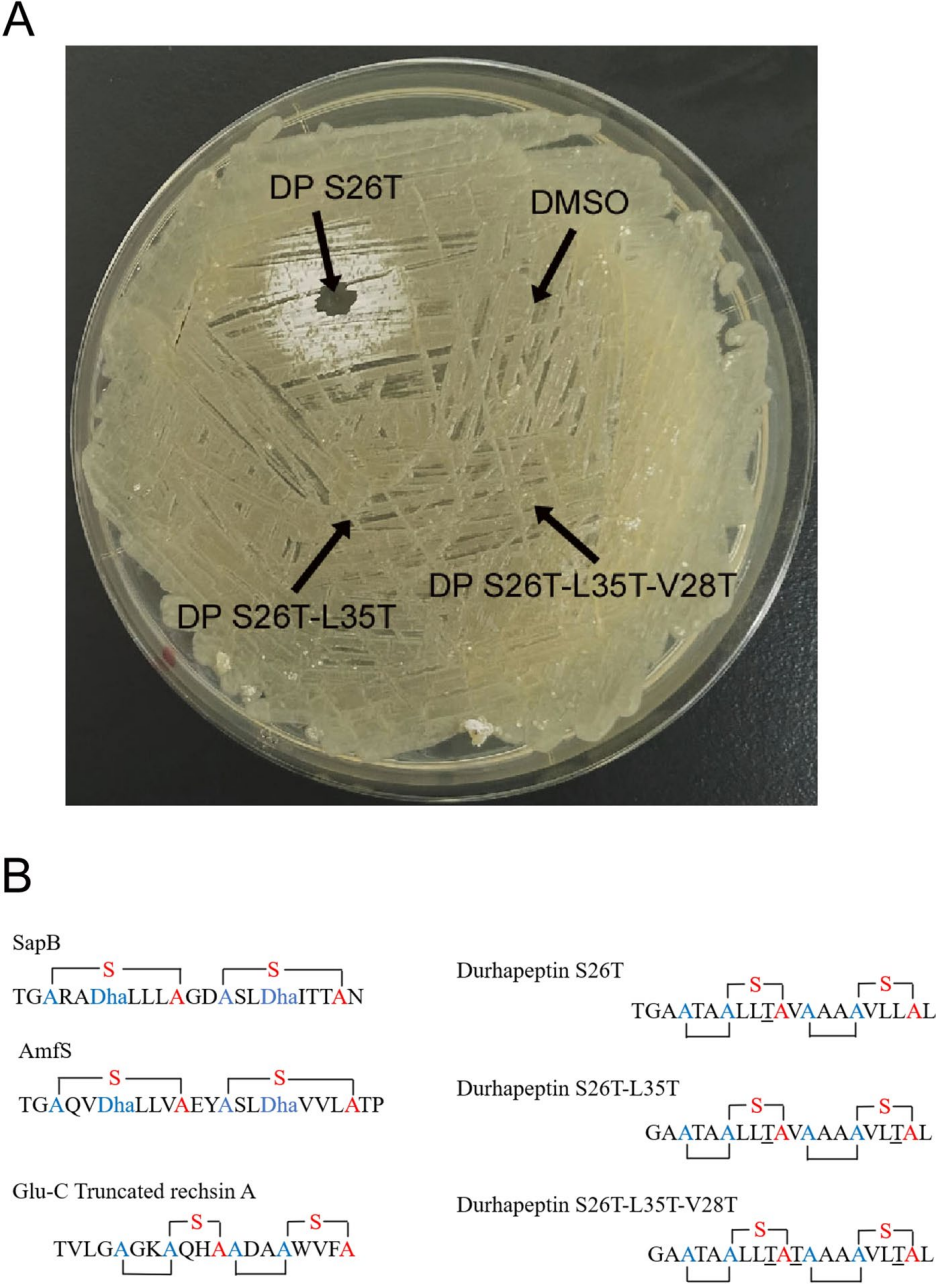
**A** Aerial hyphae inducing activities of durhapeptin (DP) S26T, S26T-L35T, and S26T-L35T-V28T to *Streptomyces asoensis*, **B** Structural comparison of the proposed thioether ring topologies between the newly synthesized durhapeptin (DP) S26T, S26T-L35T, and S26T-L35T-V28T and other known morphogenetic lanthipeptides (SapB, AmfS, and Glu-*C*-truncated rochsin A). Cys residues are indicated in red, and Ser/Thr residues are indicated in blue. The bioassay reveals that only the single-mutant variant, durhapeptin S26T, possesses the capability to induce aerial hyphae formation. This highlights the strict structural stringency required for biological signal perception

Class III lanthipeptides, such as SapB and AmfS (Fig. 4), are well-established morphogens that trigger the transition from substrate mycelium to aerial hyphae, a critical developmental step in the streptomycete life cycle (Jones and Elliot 2018). The structures of SapB and AmfS contain two lanthionine (Lan) units. Recently, rochsin A, which contains two labionin (Lab) units, was heterologously produced in *E. coli* using the BGC from *Streptomyces rochei* (Liu et al. 2024). The aerial hyphae–inducing activity of Glu-*C*–truncated rochsin A on the original strain *S. rochei* has been reported (Fig. 4); however, Glu-*C*–truncated rochsin A was not further tested. In the present study, the aerial hyphae–inducing activity of durhapeptin S26T observed in several streptomycetes harboring related biosynthetic gene clusters underscores its role as a morphogen rather than as an antimicrobial agent. Notably, durhapeptin S26T did not induce aerial hyphae formation universally but acted selectively on certain streptomycetes. This specificity suggests that morphogenetic lanthipeptides may reflect a role in kin recognition or localized developmental coordination, rather than serving as general diffusible signals across diverse streptomycetes. An intriguing observation in this study is the lack of responsiveness of the original strain, *S. durhamensis*, to durhapeptin S26T. This lack of response might be due to the structural differences between the tested S26T variant and the native wild-type peptide; other physiological factors should also be considered. For instance, durhapeptin might function as an intracellular signal rather than an extracellular one, or the exogenous peptide might have been applied at a time point that did not coincide with the specific developmental stages required for signal perception in *S. durhamensis*. The morphogen SapB has been proposed to function as a biosurfactant, decreasing the surface tension of agar medium to facilitate the formation of aerial hyphae in a manner analogous to hydrophobins in fungi (Berger and Sallada 2019; Wösten et al. 1995). Additionally, the lack of responsiveness of the original strain, *S. durhamensis*, to durhapeptin S26T may reflect differences in surface hydrophobicity or the requirement for additional regulatory components for signal perception. Furthermore, because lanthipeptide signaling and development can be highly medium-dependent, our reliance on a single medium (ISP2) might also account for the lack of activity observed in these strains, as the necessary perception machinery might remain dormant under this specific condition. Notably, the aerial hyphae–inducing activity was abolished by substitution of the hydrophobic residue Leu at position 35 with the hydrophilic residue Thr, as observed in durhapeptin S26T-L35T, which may alter hydrophobicity and thereby eliminate its function as a surfactant-like molecule to induce aerial hyphae. From a mechanistic perspective, the strong hydrophobicity of durhapeptin and its variants is noteworthy (Fig. 4). Hydrophobic surface-active peptides, such as SapB, are known to reduce surface tension at the colony–air interface, facilitating the erection of aerial hyphae. The poor solubility and aggregation-prone nature of durhapeptin are therefore consistent with a morphogenetic role that relies on physicochemical interactions at the cell surface or extracellular matrix, rather than solely on receptor-mediated signaling. These observations support the notion that class III lanthipeptide morphogens function, at least in part, by modifying the physical environment surrounding the mycelium. The widespread distribution of durhapeptin-like BGCs among streptomycetes suggests that many morphogenetic lanthipeptides remain cryptic under standard laboratory conditions. The successful heterologous expression and functional characterization of durhapeptin in this study highlight the power of genome mining combined with synthetic biology approaches to uncover hidden developmental regulators. Importantly, these findings indicate that the diversity of morphogenetic lanthipeptides may be far greater than currently appreciated, with each peptide potentially tailored to the ecological niche and developmental program of its host organism.

In conclusion, durhapeptin represents a new member of the expanding family of class III lanthipeptide morphogens. Selective aerial hyphae–inducing activity, structural similarity to known morphogens, and physicochemical properties collectively reinforce the concept that lanthipeptides play central roles in streptomycete development. Elucidating the signaling mechanisms, target specificity, and ecological functions of these peptides will be essential for a deeper understanding of morphological differentiation and multicellularity in bacteria.

## Supplementary information

Below is the link to the electronic supplementary material.

Supplementary file1 (DOCX 6969 KB)

## Data Availability

Analytical data of HPLC and CID-MS are included in supplemental material.
