# Supplementary material for: Heterologous expression reveals a cryptic morphogenetic lanthipeptide durhapeptin from Streptomyces durhamensis
Source: Antonie Van Leeuwenhoek. 2026 Jul 18;119(8):169. doi: 10.1007/s10482-026-02378-8 (PMC13380574; doi:10.1007/s10482-026-02378-8)
Supplement: Supplementary file 1 — Supplementary file1 (DOCX 6969 KB) [file 10482_2026_2378_MOESM1_ESM.docx]

**Supplemental Information**

Title: Heterologous expression reveals a cryptic morphogenetic lanthipeptide durhapeptin from *Streptomyces durhamensis*

Authors: Marino Tsugimoto^1^, Ryo Kobayashi^1^, Chanaphat Thetsana^2^, Hiroyuki Nakagawa,^3^ Tomohiro Furukawa,^4^ and Shinya Kodani^1,2,5^*

Affiliations: ^1^Graduate School of Integrated Science and Technology, Shizuoka University, Shizuoka 422-8529, Japan; ^2^Graduate School of Science and Technology, Shizuoka University, Shizuoka 422-8529, ^3^Research center for Advanced Analysis, National Agriculture and Food Research Organization (NARO), Ibaraki 305-8642, Japan; ^4^Institute of Food Research, NARO, Ibaraki 305-8642, Japan; ^5^College of Agriculture, Academic Institute, Shizuoka University, Shizuoka, 422-8529 Japan

*To whom correspondence should be addressed: Shinya Kodani, College of Agriculture, Academic Institute, Shizuoka University, 836 Ohya, Suruga-ku, Shizuoka 422-8529 Japan, Tel/Fax; +81(54)238-5008, E-mail; [kodani.shinya@shizuoka.ac.jp](mailto:kodani.shinya@shizuoka.ac.jp), ORCID:0000-0002-6792-1184

**Contents**

**Table S1.** Deduced functions of the proteins encoded in the *dur* cluster and its neighboring genes.

**Table S2.** DNA sequences of primers used in this study

**Table S3.** Product ions on CID-MS analysis of TFA-treated durhapeptin S26T-L35T-V28T

**Table S4**. Aerial hyphae inducing activities of durhapeptin S26T (5 μg) to streptomycetes

**Fig. S1.** Sequence of durKC and durA for pET29b, red letter: durA, green: SD sequence, light blue: durKC, underlined letter: restriction enzyme recognition site (NdeI and KpnI).

**Fig. S2.** Plasmid map for pET29b-13441: the insert was integrated into multicloning sites (NdeI and KpnI) to use T7 promoter system

**Fig. S3.** A) HPLC analysis of MeOH extract of *E. coli* BL21(DE3) harboring pET29b-13441 (arrow: durhapeptin); the structural identity of the indicated peak at ~13 min was confirmed by ESI-MS analysis (Fig. S4). B) HPLC analysis of MeOH extract of *E. coli* BL21(DE3) harboring pET-29b(+).

**Fig. S4.** ESI-MS analysis of durhapeptin. Note: A corresponding ESI-MS profile for the control strain could not be generated due to the complete absence of an eluting peak at the corresponding retention time in the HPLC profile (see Fig. S3B).

**Fig. S5.** HPLC analysis of MeOH extract of E. coli BL21(DE3) harboring pET29b-13441-S26T (arrow: durhapeptin S26T)

**Fig. S6.** ESI-MS analysis of durhapeptin S26T

**Fig. S7.** HPLC analysis of MeOH extract of E. coli BL21(DE3) harboring pET29b-13441-S26T-L35T (arrow: durhapeptin S26T-L35T)

**Fig. S8.** ESI-MS analysis of durhapeptin S26T-L35T

**Fig. S9.** HPLC analysis of MeOH extract of E. coli BL21(DE3) harboring pET29b-13441-S26T-L35T-L24T (arrow: durhapeptin S26T-L35T-L24T)

**Fig. S10.** ESI-MS analysis of durhapeptin S26T-L35T-L24T

**Fig. S11.** HPLC analysis of MeOH extract of E. coli BL21(DE3) harboring pET29b-13441-S26T-L35T-L25T (arrow: durhapeptin S26T-L35T-L25T)

**Fig. S12.** ESI-MS analysis of durhapeptin S26T-L35T-L25T

**Fig. S13.** HPLC analysis of MeOH extract of E. coli BL21(DE3) harboring pET29b-13441-S26T-L35T-V28T (arrow: durhapeptin S26T-L35T-V28T)

**Fig. S14.** ESI-MS analysis of durhapeptin S26T-L35T-V28T

**Fig. S15.** HPLC analysis of TFA treated durhapeptin S26T-L35T-V28T (arrow: partially hydrolyzed durhapeptin S26T-L35T-V28T)

**Fig. S16.** ESI-MS analysis of TFA treated durhapeptin S26T-L35T-V28T

**Fig. S17.** CID-MS analysis of TFA treated S26T-L35T-V28T-TFA (m/z 100-2000)

**Fig. S18.** Enlarged CID-MS chart of TFA treated durhapeptin S26T-L35T-V28T (apply voltage 80 eV, m/z 750-786)

**Fig. S19.** Enlarged CID-MS chart of TFA treated durhapeptin S26T-L35T-V28T (apply voltage 80 eV, m/z 805-870)

**Fig. S20.** Enlarged CID-MS chart of TFA treated durhapeptin S26T-L35T-V28T (apply voltage 80 eV, m/z 876-906)

**Fig. S21.** Enlarged CID-MS chart of TFA treated durhapeptin S26T-L35T-V28T (apply voltage 80 eV, m/z 986-1008)

**Fig. S22.** aerial hyphae inducing activity of durhapeptin S26T to Streptomyces asoensis (red arrow: durhapeptin S26T 5 μg, black arrow: negative control DMSO)

**Fig. S23.** aerial hyphae inducing activity of durhapeptin S26T to Streptomyces maremycinicus (red arrow: durhapeptin S26T 5 μg, black arrow: negative control DMSO)

**Fig. S24.** aerial hyphae inducing activity of durhapeptin S26T to Streptomyces humidus (red arrow: durhapeptin S26T 5 μg, black arrow: negative control DMSO)

**Table S1.** Deduced functions of the gene encoded in the *dur* cluster and its neighboring genes.

| Gene | Accession number | aa | Annotated function |
| --- | --- | --- | --- |
| ORF1 | WP_031172265 | 91 | MULTISPECIES: hypothetical protein |
| ORF2 | WP_031172267 | 820 | MULTISPECIES: SpoIIE family protein phosphatase [*Streptomyces*] |
| *durKC* | WP_031172270 | 870 | Class III lanthipeptide synthetase LanKC |
| *durA* | WP_031172271 | 37 | SapB/AmfS family lanthipeptide |
| *durT* | WP_051927124 | 597 | ABC transporter ATP-binding protein [*Streptomyces durhamensis*] |
| ORF3 | WP_280921914 | 678 | ATP-binding cassette domain-containing protein [*Streptomyces durhamensis*] |
| ORF4 | WP_031172277 | 202 | Response regulator transcription factor |

**Table S2.** DNA sequences of primers used in this study

| Primer name | DNA Sequence (5’ to 3’) |
| --- | --- |
| 13441-S26T-F | CCTGCTGACCTGTGTTTCTGCAGCGTCTGTGCGCTGTGTСT |
| 13441-S26T-R | AACACAGGTCAGCAGGGAAGCAGTGGACGCACCGGTGTGAGT |
| 13441-S26T-L34T-F | GTCTGTGACGCTGTGTCTGTAATGAGGAGATATAA |
| 13441-S26T-L34T-R | ACACAGCGTCACAGACGCTGCAGAAACACAGGTCA |
| 13441-S26T-L35T-F | TGTGCTGACGTGTCTGTAATGAGGAGATATAACTA |
| 13441-S26T-L35T-R | ACAGACACGTCAGCACAGACGCTGCAGAAACACAG |
| 13441-S26T-L37T-F | GCTGTGTACGTAATGAGGAGATATAACTATGGACA |
| 13441-S26T-L37T-R | CTCATTACGTACACAGCAGCACAGACGCTGCAGAA |
| 13441-S26T-L35T-L24T-F | TGCTTCCACGCTGACCTGTGTTTCTGCAGCGTCT |
| 13441-S26T-L35T-L24T-R | GGTCAGCGTGGAAGCAGTGGACGCACC |
| 13441-S26T-L35T-L25T-F | TTCCCTGACGACCTGTGTTTCTGCAGCGTCTGTG |
| 13441-S26T-L35T-L25T-R | ACAGGTCGTCAGGGAAGCAGTGGACGCAC |
| 13441-S26T-L35T-V28T-F | GACCTGTACTTCTGCAGCGTCTGTGCTGACGTGT |
| 13441-S26T-L35T-V28T-R | TGCAGAAGTACAGGTCAGCAGGGAAGCAGTGGACG |

**Table S3.** Product ions on CID-MS analysis of TFA-treated durhapeptin S26T-L35T-V28T


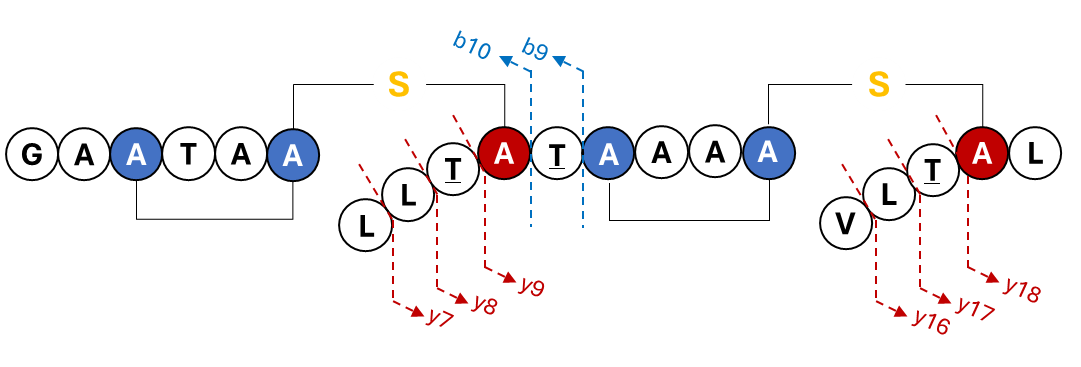


| **fragment ion** | **ion** | **predicted mass** | **observed mass** |
| --- | --- | --- | --- |
| y7 | +2 | 861.4 | 860.9 |
| y8 | +2 | 804.7 | 804.3 |
| y9 | +2 | 754.3 | 753.8 |
| y16 | +2 | 761.3 | 760.8 |
| y17 | +2 | 811.9 | 811.3 |
| y18 | +2 | 868.4 | 867.9 |
| b9 | +1 | 988.4 | 988.4 |
| b10 | +1 | 887.4 | 887.4 |

| **Table S4**. Aerial hyphae inducing activities of durhapeptin S26T (5 μg) to *Streptomycetes* | | |
| --- | --- | --- |
| Strain name | activity |  |
| *Streptomyces durhamensis* | - |  |
| *Streptomyces filipinensis* | - |  |
| *Streptomyces hygroscopicus* | + |  |
| *Streptomyces djakartensis* | - |  |
| *Streptomyces siamensis* | - |  |
| *Streptomyces humidus* | + |  |
| *Streptomyces alanosinicus* | - |  |
| *Streptomyces maremycinicus* | + |  |
| *Streptomyces blastmyceticus* | - |  |
| *Streptomyces hawaiiensis* | - |  |
| *Streptomyces umbrinus* | - |  |
| *Streptomyces asoensis* | + |  |
| *Streptomyces echinoruber* | - |  |
| *Streptomyces cinereospinus* | - |  |
| *Streptomyces mobaraensis* | - |  |
| *Streptomyces davaonensis* | - |  |
| *Streptomyces viridochromogenes* | - |  |

+: induced, -: not induced

**Fig. S1.** Sequence of *durKC* and *durA* for pET29b, red letter: *durA*, green: SD sequence, light blue: *durKC*, underlined letter: restriction enzyme recognition site (NdeI and KpnI).

CATATGGCCCTGCTGGATCTGCAGACCCTGGAATCTGATGAATATACTCACACCGGTGCGTCCACTGCTTCCCTGCTGACCTGTGTTTCTGCAGCGTCTGTGCTGCTGTGTCTGTAATGAGGAGATATAACTATGGACAAACGTTACGAAGTCTATGCGCTGGCGGATGGTCATTTTTACGACACTCCGGATCGTCTGCCAGGTGCAGATGGTGCACCGGCTGCTCTGTACGCAACTGCACGTCGTACTGTACCGGAGGGTTGGCACGCAGCACGTTCTGGTGACTGGCTGACCCTGACCCCAGTGGATGCTGATGGTGCTCCGCTGCCTTCTCCGGCTCAAGGTTGGAAAATCCACTCCTCTGCAACCGCTGCGAACGCAGAACGCATTGCTGATATCGTGTGGGACTACTGCGTTCCTCGTCGTATCCCGTTCAAATTCGTGCCGGGTCCACACCTGCTGCATCTGCGTAATGCGAAATATGCCGGTCGTGACACTTCCGGCAAGTTCGTGACCGTGTACCCTGCCGACGAAGAACAGCTGCAGCAGGTTCTGCGTGAACTGGGTGCTCTGCTGCAGGGTTTCGAAGGTCCATATATCCTGACCGATCTGCGTTGGCACGATGGCCCGCTGTACGTACGTTACGGTGCCTTTGCTCGTCGCTATGTGGTTGATGAGCGTGGTTCTCTGGTTCCGGCAGTAGCTGATGGTACTGGTCGTCTGGTTCCGGATCGTCGTGCGCCAAGCTTCCAGGTTCCGGAGTGGGTAACCCTGCCAGAATTTCTGCGTCCGCACCTGGACGCTCGTAACACCACTACCGTTGGCGAGCTGCCGTACCGTATCGAAAAAGCGCTGCACTTCTCCAACGGCGGTGGTGTATACACCGGTACGGACACTCGTGATGGCTGTCGTGTGGTTCTGAAAGAAGCACGTCCTCATGCTGGTCTGGCAGCGGATGGCGCAGATGCAGTAGCTCGTCTGGAACGTGAAAAGGCTGCTCTGGAACGTGTGGCCGGTACTGGTGTAGTACCGGAAGTTCGCGACTGGTTTACCCTGGGTGAACACCGCTTCCTGGTTATGGACCACCTGGCTGGCCGTCCTCTGAACTCCTTCTTCGCTGAACGTCACCCACTGCTGACTGCTGATCCAGATCCGGCTGGTGTTGCAGCATATACCGCATGGGCACTGCGCATCAACGCAGCGGTGGAACAGGCTGTGGCTGCTGTACATGCTCGTGGCCTGGTTTTTAACGATCTGCATGTTTTCAACATTATGGTGGCCGAAGATGAGCAGAGCGTGCACCTGATTGATTTTGAGGCAGCTGCTCCTGCTGAAGCTAATGCGCGTCAGACTGTGGCGCATCCAGGTTTCTTCGCACCACCGGATCGTCGTGGCCCGGATGTAGATCGTTATGCTCTGGCGTGTCTGCGTCTGGCACTGTTCCTGCCGGTTACCACCCTGTTCGTTGTTGACCGTGGCAAAGCGGCACACCTGGCAGAAGTTATCGCGGAACAGTTCCCGGATGTACCGCGTGCGTTCCTGGACGAAGCTGTAGCAGAAATCACGCGTGATACTGCAGGTGGTCGTCGTTCTCGTCCAGCTCCACCTGTTGTACCGGGTGACTGGCCGTACTCTCGTGATTCCATGGTAAAAGCTATTCTGGCCTCCGCTACCCCGGAGCGTGATGATCGTCTGTTCCCTGGTGACATTGCACAGTTTTCTGATGGTGGTGGTCTGGGTCTGGCACACGGTGCTGCTGGTGTTCTGCACGCACTGCAAGCTGCTGGTGCAGGTCGCTACGACGAAGGTGAACATTGGCTGCTGGCCCATACGGATCCTCCACCTCCAGGTACTCCGCTGGGTCTGTACGATGGTCTGGCCGGTGTTGCCCTGGTTCTGGATCAGCTGGGTCATCGTCAGCGTGCTCTGGACCTGACCGATGGTGTGCTGCGCGAAAACTGGCGCTCCCTGGCTTCTGATCTGCAGGGTGGTCTGGCTGGTCTGGGTCTGGTTCTGGGTCGTCTGGCTGATACGACTGGCGAAAGCGCTCTGCGTCAACACGCTGCTGAAGCAGCTGATATTCTGGTTCGTCGTCTGGCAGAACCGCTGCCGGATACTCCACGTCGTCGTGCTGGTCTGCTGCGTGGTGCTACTGGTCCTGCACTGTTTCTGCTGCGCTGGTACGAACAGACTGGCGAAGATCGCTTTCTGACTGCGGCTGCAGAAGCTCTGCGTCGTGATCTGGACTGTTGTGTGACCCAGGAAGCTGGCGGTGGCCTGGAAGTTGATGAAGGTTGGCGTACTCTGCCGTATCTGGGTGATGGTTCCGCTGGTATCGGCCTGGTACTGGACGACTACCTGGCACACGCGGATGACGGTGAATTTGAACGTGCCCGTGCTGGCATTCTGACCGCTGCAACCTCTCGTTTCTACGCTCAGCCGGGTCTGTTTCAAGGCCGTGCTGGCATGATTCTGCATCTGGCCCGTTCTACCGCTCCGGGTGCTACTCGTGAACGTCTGCGCCAGCAGATTGCCGGTCTGGGTTGGTTCGCCATGGACTACCAGGGTCAGCTGGCGTTTCCGGGTCATCAGATGATGCGTCTGTCTATGGATCTGGCTACCGGCACTGCAGGTTGTCTGCTGGCACTGGCAGCAGCTCGTGATGACGCTCGTACTGCTCATCTGCCATTTCTGGCTCCGCCACCAGCTGCTCCAACTCGTGGTTCCGCACCAACTAAACCGTAAGGTACC

**Fig. S2.** Plasmid map for pET29b-13441: the insert was integrated into multicloning sites (NdeI and KpnI) to use T7 promoter system


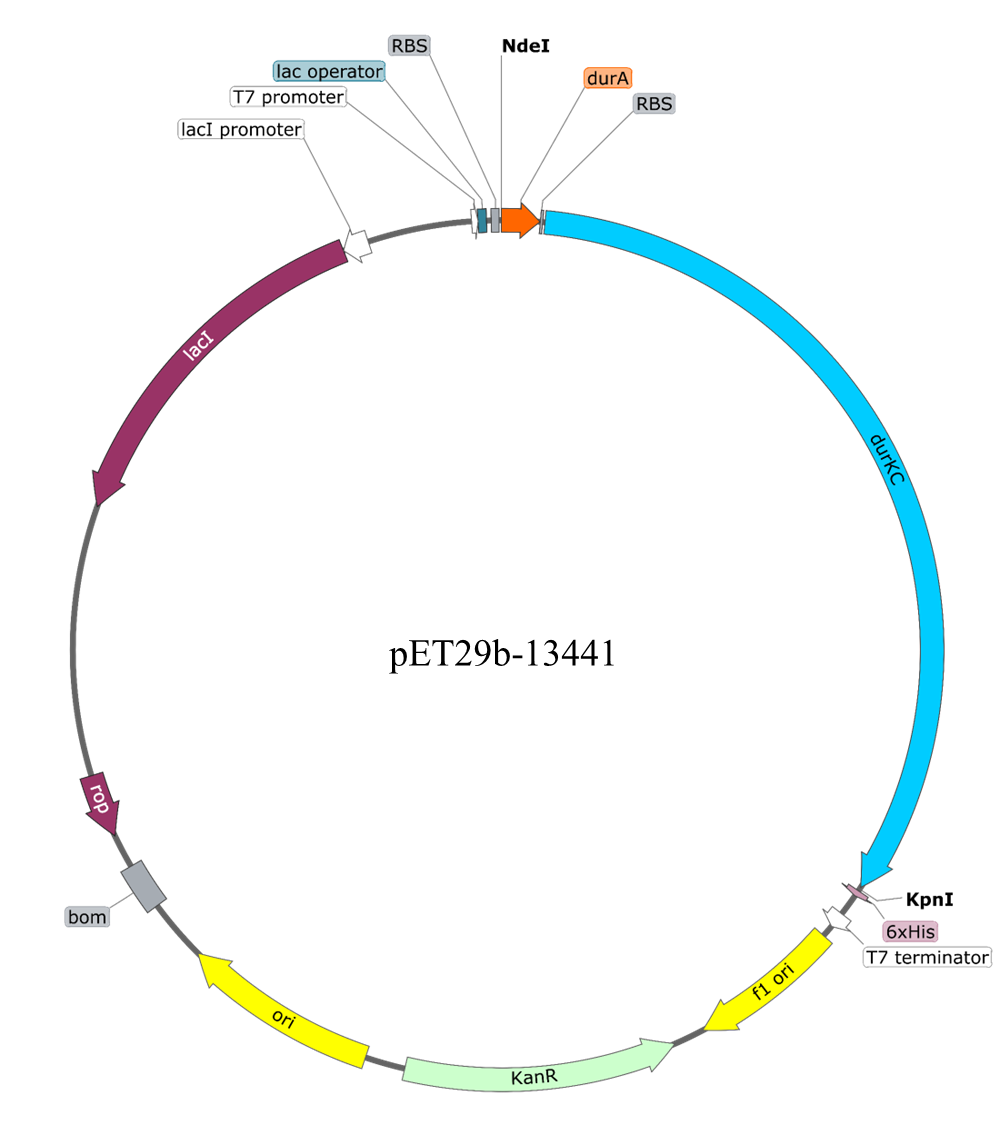


**Fig. S3.** A) HPLC analysis of MeOH extract of *E. coli* BL21(DE3) harboring pET29b-13441 (arrow: durhapeptin); the structural identity of the indicated peak at ~13 min was confirmed by ESI-MS analysis (Fig. S4). B) HPLC analysis of MeOH extract of *E. coli* BL21(DE3) harboring pET-29b(+).

A


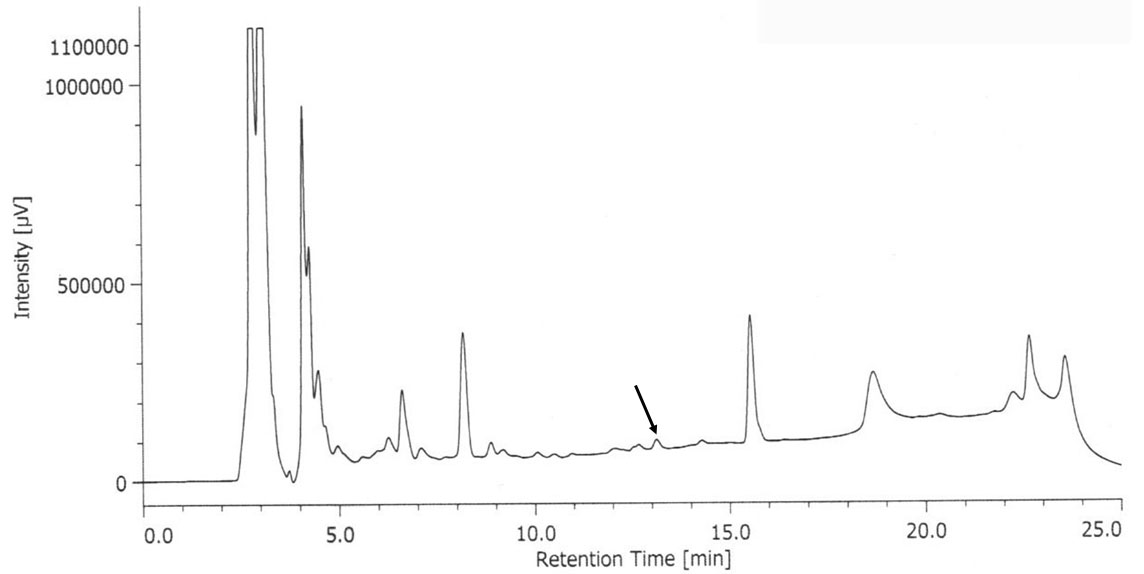


B


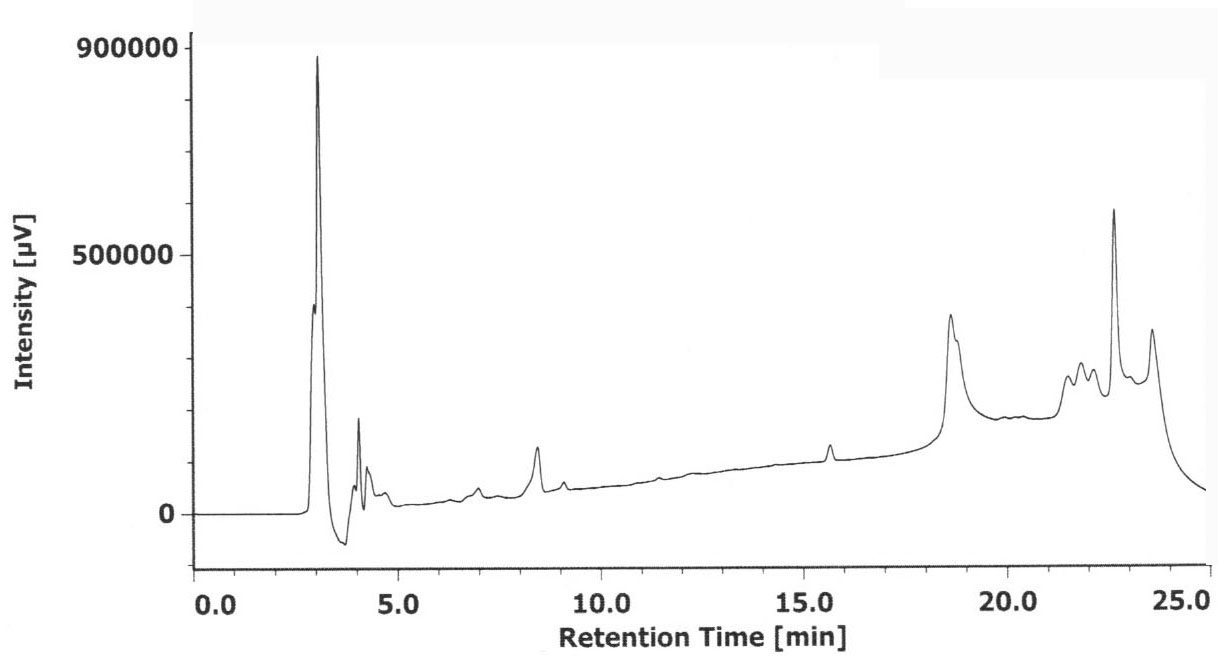


**Fig. S4.** ESI-MS analysis of durhapeptin. Note: A corresponding ESI-MS profile for the control strain could not be generated due to the complete absence of an eluting peak at the corresponding retention time in the HPLC profile (see Fig. S3B).


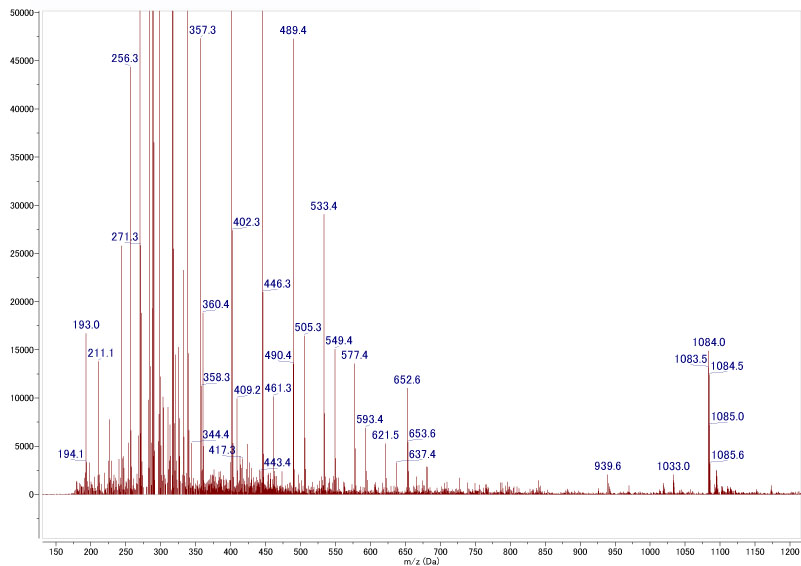


**Fig. S5.** HPLC analysis of MeOH extract of *E. coli* BL21(DE3) harboring pET29b-13441-S26T (arrow: durhapeptin S26T)

**Fig. S6.** ESI-MS analysis of durhapeptin S26T


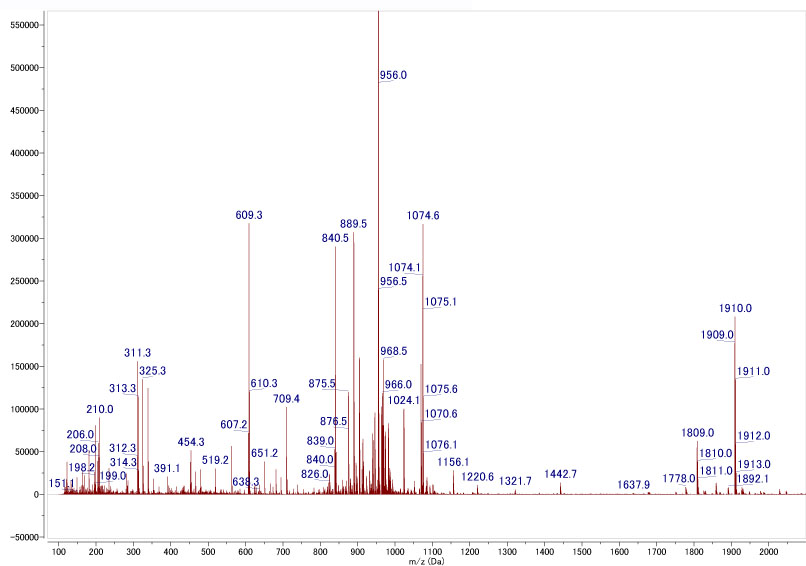


**Fig. S7.** HPLC analysis of MeOH extract of *E. coli* BL21(DE3) harboring pET29b-13441-S26T-L35T (arrow: durhapeptin S26T-L35T)

**Fig. S8.** ESI-MS analysis of durhapeptin S26T-L35T


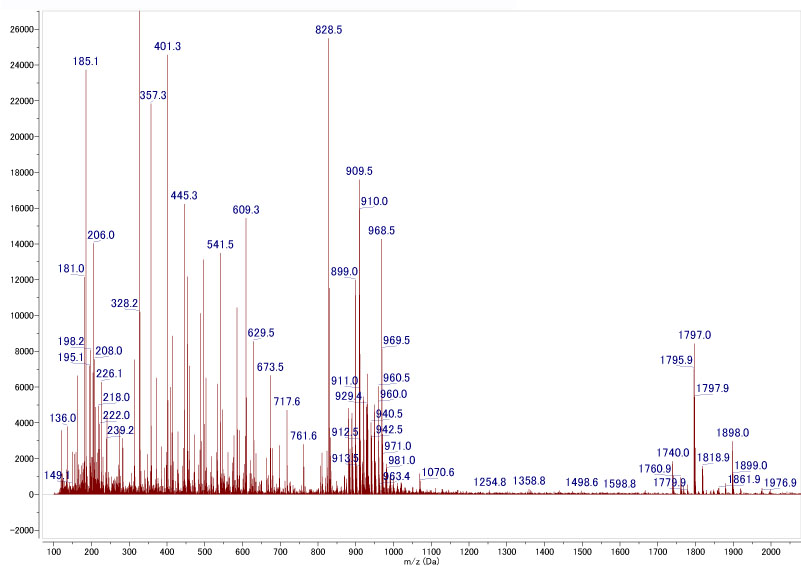


**Fig. S9.** HPLC analysis of MeOH extract of *E. coli* BL21(DE3) harboring pET29b-13441-S26T-L35T-L24T (arrow: durhapeptin S26T-L35T-L24T)

**Fig. S10.** ESI-MS analysis of durhapeptin S26T-L35T-L24T


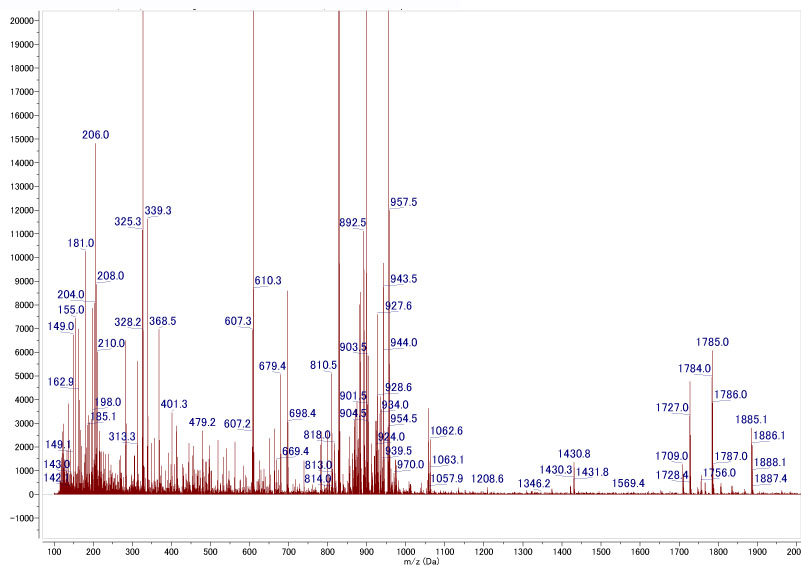


**Fig. S11.** HPLC analysis of MeOH extract of *E. coli* BL21(DE3) harboring pET29b-13441-S26T-L35T-L25T (arrow: durhapeptin S26T-L35T-L25T)

**Fig. S12.** ESI-MS analysis of durhapeptin S26T-L35T-L25T


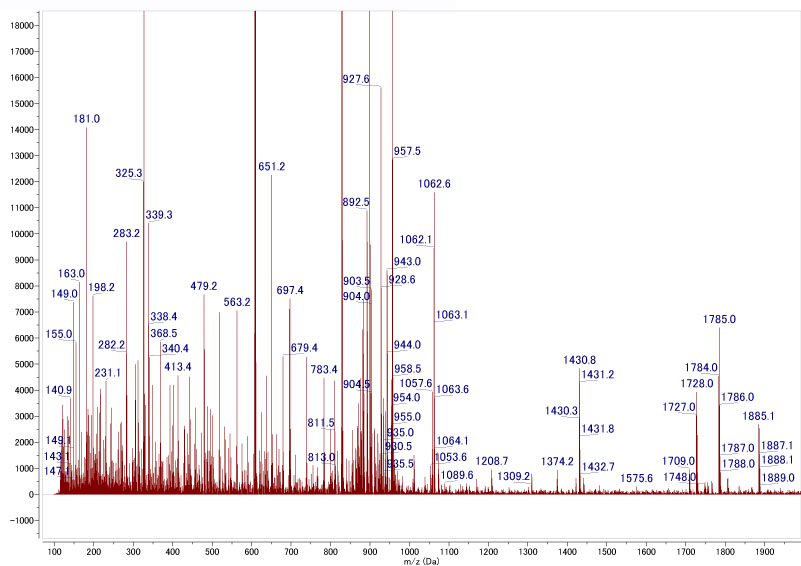


**Fig. S13.** HPLC analysis of MeOH extract of *E. coli* BL21(DE3) harboring pET29b-13441-S26T-L35T-V28T (arrow: durhapeptin S26T-L35T-V28T)

**Fig. S14.** ESI-MS analysis of durhapeptin S26T-L35T-V28T


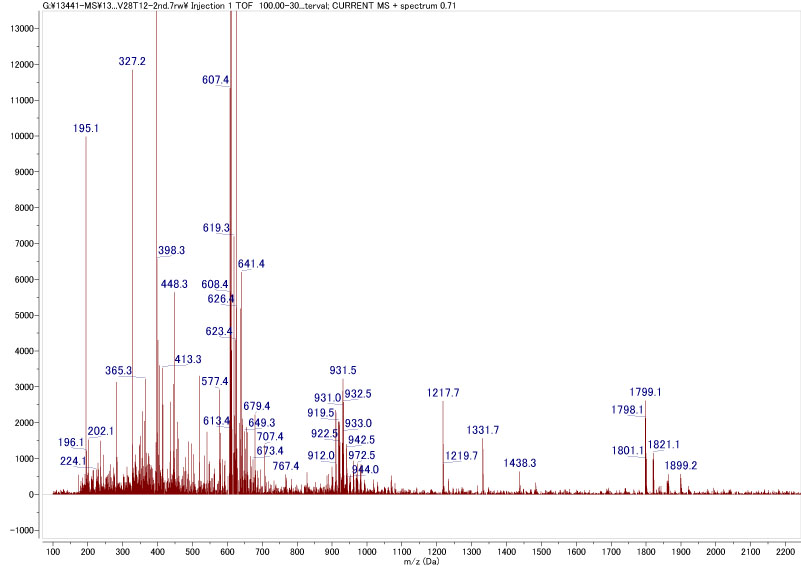


**Fig. S15.** HPLC analysis of TFA treated durhapeptin S26T-L35T-V28T (arrow: partially hydrolyzed durhapeptin S26T-L35T-V28T)

**Fig. S16.** ESI-MS analysis of TFA-treated durhapeptin S26T-L35T-V28T


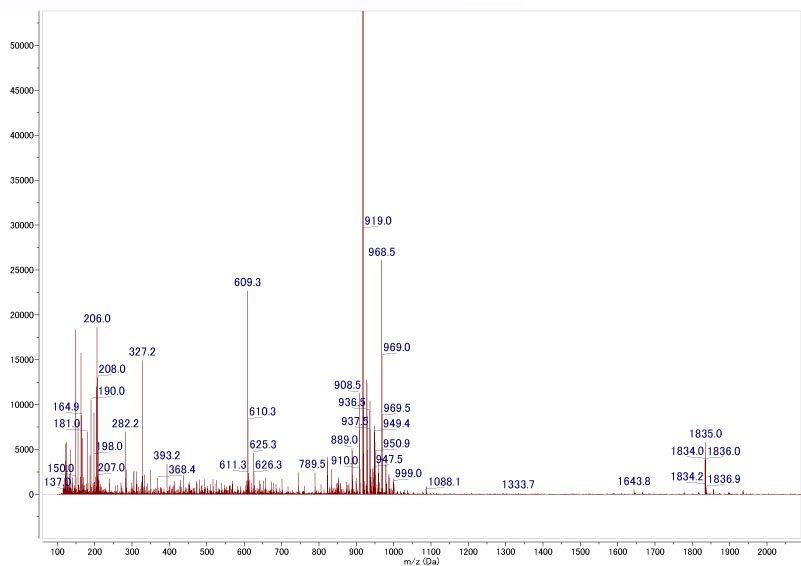


**Fig. S17.** CID-MS analysis of TFA treated S26T-L35T-V28T-TFA (m/z 100-2000)


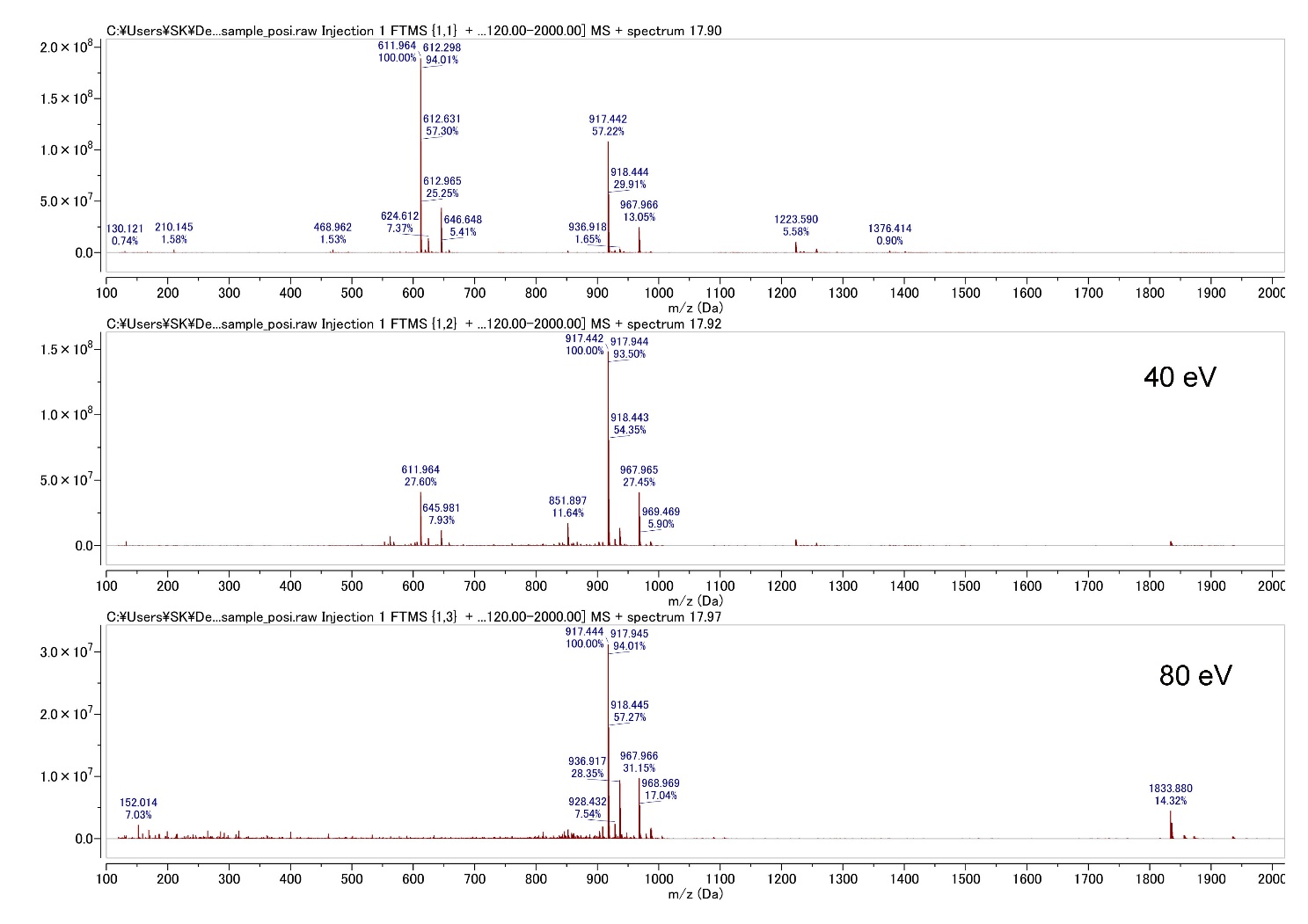


**Fig. S18.** Enlarged CID-MS chart of TFA treated durhapeptin S26T-L35T-V28T (apply voltage 80 eV, m/z 750-786)


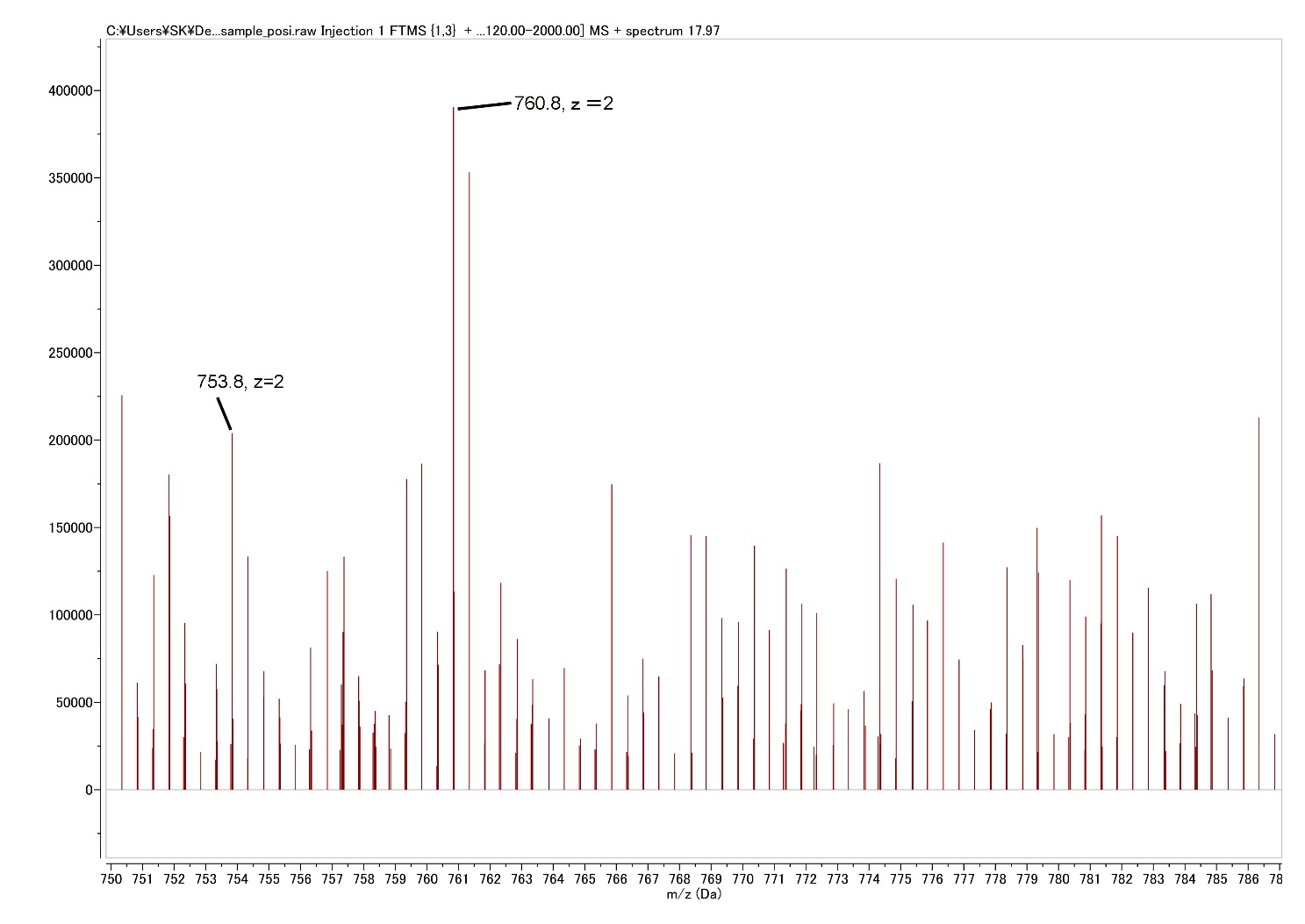


**Fig. S19.** Enlarged CID-MS chart of TFA treated durhapeptin S26T-L35T-V28T (apply voltage 80 eV, m/z 805-870)


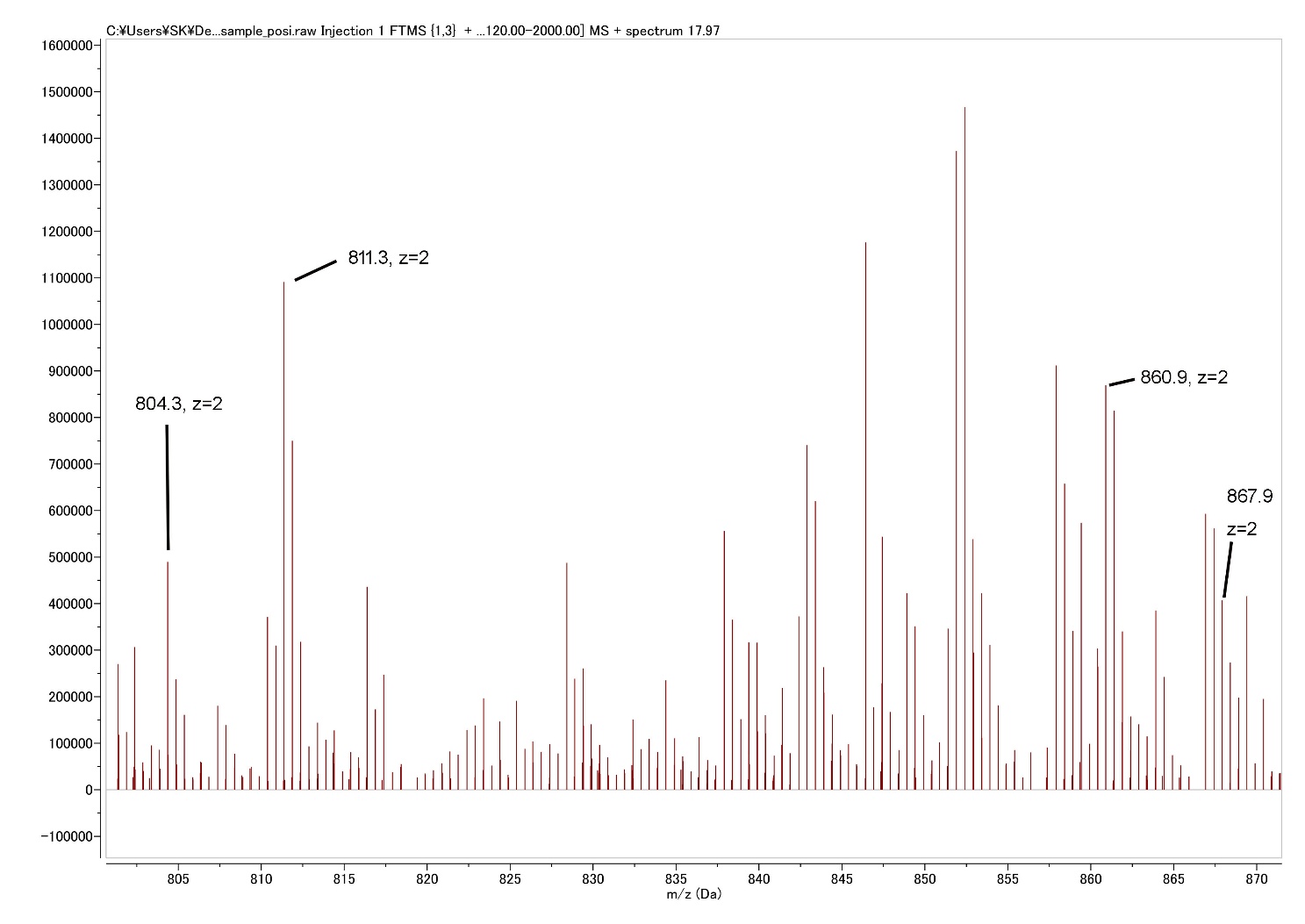


**Fig. S20.** Enlarged CID-MS chart of TFA treated durhapeptin S26T-L35T-V28T (apply voltage 80 eV, m/z 876-906)


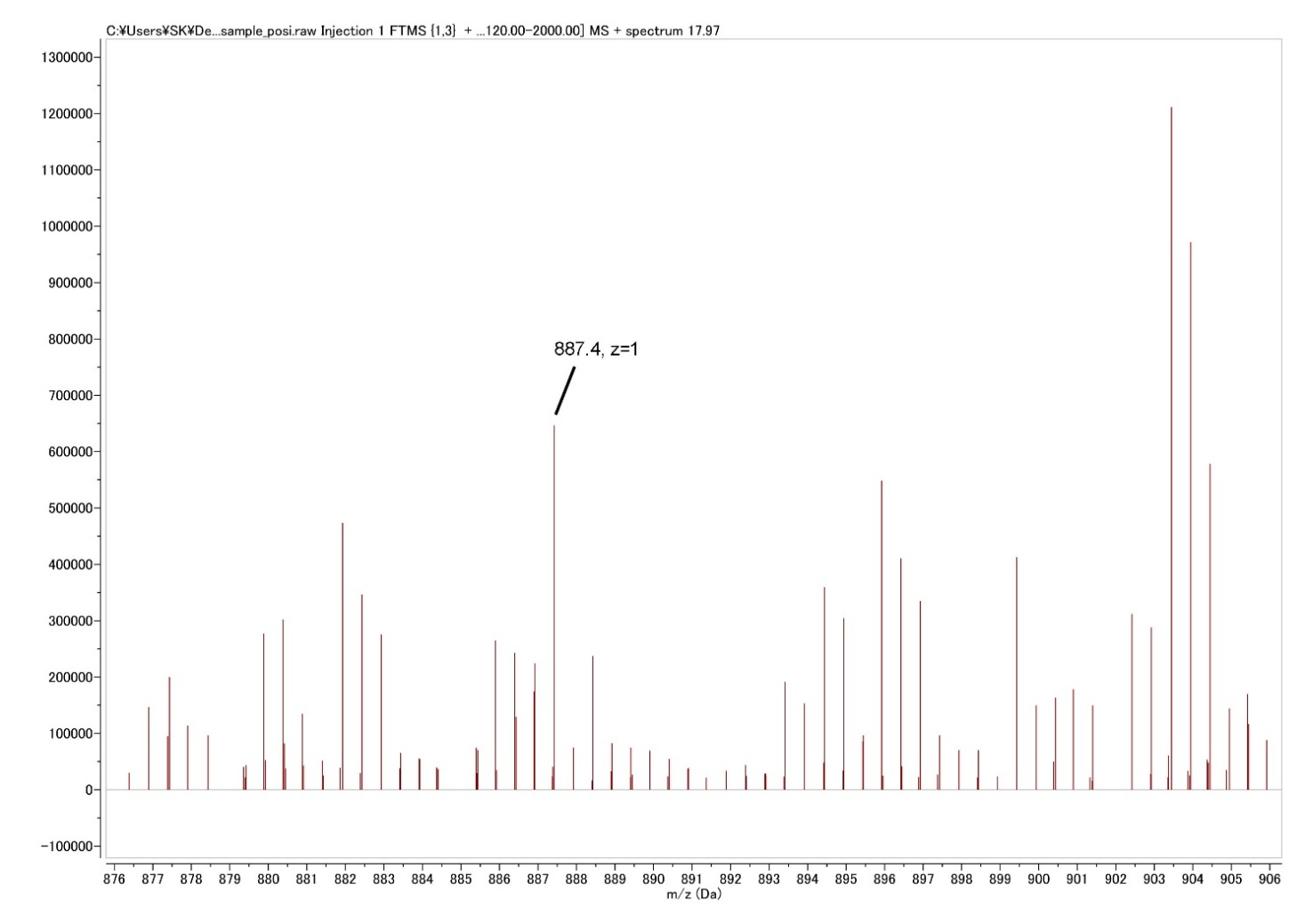


**Fig. S21.** Enlarged CID-MS chart of TFA treated durhapeptin S26T-L35T-V28T (apply voltage 80 eV, m/z 986-1008)


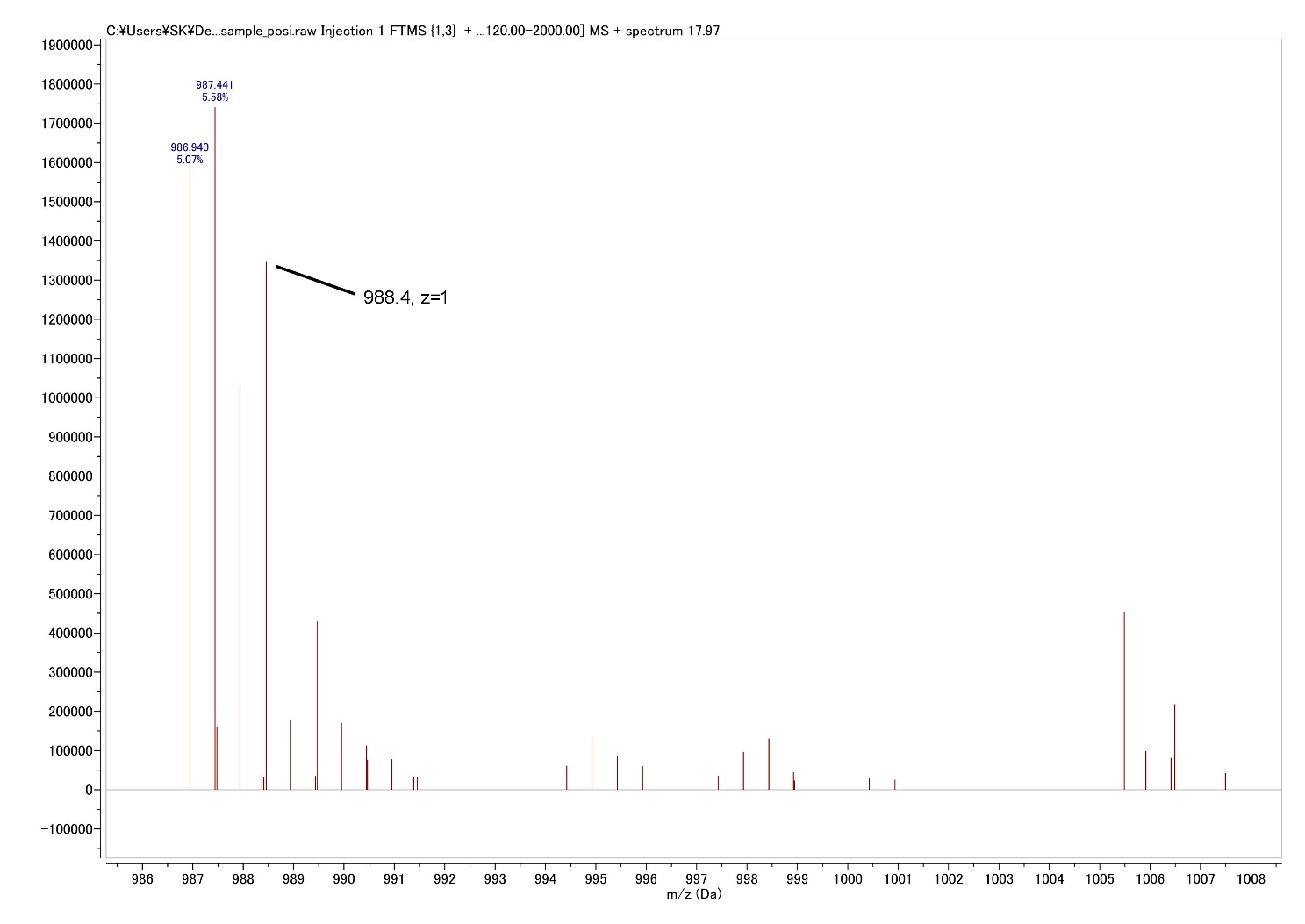


**Fig. S22.** Aerial hyphae inducing activity of durhapeptin S26T to *Streptomyces asoensis* (red arrow: durhapeptin S26T 5mg, black arrow: negative control DMSO)

**Fig. S23.** Aerial hyphae inducing activity of durhapeptin S26T to *Streptomyces maremycinicus* (red arrow: durhapeptin S26T 5mg, black arrow: negative control DMSO)

**Fig. S24.** Aerial hyphae inducing activity of durhapeptin S26T to *Streptomyces humidus* (red arrow: durhapeptin S26T 5mg, black arrow: negative control DMSO)
